# St. Mary’s Hospital

**Published:** 1852-11

**Authors:** 


ST. MARY'S HOSPITAL. *Physicians*—Drs. Alderson, Chambers, and Sibson. *Surgeons*—Messrs. Coulson, Lane, and Ure. *Physician Accoucheur*—Dr. Tyler Smith. *Surgeon Accoucheur*—Mr. Baker Brown. *Ophthalmic Surgeon*—Mr. W. Cooper. *Aural Surgeon*—Mr. Toynbee. *Assistant Physicians*—Drs. H. Jones, Sieveking, and Markham. *Assistant Surgeons*—Messrs. Smith, Walton, and Lane. *Dental Surgeon*—Mr. Nasmyth.

